# Structure and nucleic acid binding properties of KOW domains 4 and 6–7 of human transcription elongation factor DSIF

**DOI:** 10.1038/s41598-018-30042-3

**Published:** 2018-08-03

**Authors:** Philipp K. Zuber, Lukas Hahn, Anne Reinl, Kristian Schweimer, Stefan H. Knauer, Max E. Gottesman, Paul Rösch, Birgitta M. Wöhrl

**Affiliations:** 1 Universität Bayreuth, Lehrstuhl Biopolymere, Universitätsstr. 30, D-95447 Bayreuth, Germany; 2 Forschungszentrum für Bio-Makromoleküle, Universitätsstr. 30, D-95447 Bayreuth, Germany; 3 Department of Microbiology and Immunology, Columbia University, New York, NY USA

## Abstract

The human transcription elongation factor DSIF is highly conserved throughout all kingdoms of life and plays multiple roles during transcription. DSIF is a heterodimer, consisting of Spt4 and Spt5 that interacts with RNA polymerase II (RNAP II). DSIF binds to the elongation complex and induces promoter-proximal pausing of RNAP II. Human Spt5 consists of a NusG N-terminal (NGN) domain motif, which is followed by several KOW domains. We determined the solution structures of the human Spt5 KOW4 and the C-terminal domain by nuclear magnetic resonance spectroscopy. In addition to the typical KOW fold, the solution structure of KOW4 revealed an N-terminal four-stranded β-sheet, previously designated as the KOW3-KOW4 linker. In solution, the C-terminus of Spt5 consists of two β-barrel folds typical for KOW domains, designated KOW6 and KOW7. We also analysed the nucleic acid and RNAP II binding properties of the KOW domains. KOW4 variants interacted with nucleic acids, preferentially single stranded RNA, whereas no nucleic acid binding could be detected for KOW6-7. Weak binding of KOW4 to the RNAP II stalk, which is comprised of Rpb4/7, was also detected, consistent with transient interactions between Spt5 and these RNAP II subunits.

## Introduction

Eukaryotic transcription catalysed by the enzyme RNA polymerase II (RNAP II) is tightly regulated by a variety of mechanisms. An important and widespread regulatory step is promoter proximal transcriptional pausing, which introduces an early block to RNAP II elongation after 20 to 70 transcribed bases. Participation of the general transcription factor DRB (5,6,-dichloro-1-β-D-ribofuranosylbenzimidazole) sensitivity inducing factor (DSIF) in this transcription rate limiting step is required for normal RNA synthesis^1–4^.

Human DSIF is a heterodimer composed of a 14 kDa (hSpt4) and a 120 kDa (hSpt5) subunit, the human homologues of Spt4 and Spt5 of *Saccharomyces cerevisiae*^2,5^. The hSpt5 subunit contains a region that is homologous to the N-terminal domain of the bacterial transcription factor NusG (NGN). NusG/Spt5 proteins are conserved in all three kingdoms of life^6^. Bacterial NusG and the archaeal Spt5 proteins are composed of the NGN-domain and a flexibly-connected C-terminal Kyrpides-Ouzounis-Woese (KOW) domain. Eukaryotic Spt5 proteins harbour several KOW domain copies whose functions have not been studied in full detail^7,8^. The function of the NGN is conserved in bacteria, *archaea*, and eukaryotes. In eukaryotes, the NGN domain binds to the Rpb1 and Rpb2 subunits of RNAP II forming a processivity clamp^9,10^. It thereby locks the nucleic acids with the RNAP in a closed and pause-resistant state^11,12^. Spt4, which is not present in bacteria, interacts with Spt5 *via* the NGN-domain.

Additionally, eukaryotic Spt5 includes an acidic region at its N-terminus and carries two C-terminal repeat (CTR) regions that can be phosphorylated by the positive transcription elongation factor pTEFb. Phosphorylated DSIF activates RNAP II elongation^13^. Whereas KOW1-5 domains have been detected in all analysed eukaryotes, the region downstream of the CTRs can only be found in metazoan and plant Spt5^14,15^.

Although the structure of the mammalian RNAP II elongation complex has been determined, the precise functions of the different KOW domains of DSIF as well as that of the N-terminal acidic regions remain elusive^9,10^. hSpt5 is known to interact with RNAP II not only *via* the NGN but also through the region harbouring the KOW motifs. The KOW1 domain is flexible and binds between the clamp and the RNA exit tunnel of RNAP II^9^. Crosslinks and protein mass finger prints demonstrated an interaction between the C-terminal region of hSPT5 and Rpb1. The position of DSIF lies over the active centre cleft in the clamp domain of RNAP II^9^.

A cryo-electron microscopy (EM) structure of the mammalian RNAP II/DSIF complex and cross-linking experiments with Spt4/5 from *Saccharomyces cerevisiae* show that the Spt5 KOW4 interacts with RNAP II subunits Rpb4 and Rpb7^10,16^. In *archaea*, RpoE and RpoF are homologous to Rpb7 and Rpb4, respectively, and have similar functions^17,18^. Rpb4 is suggested to function mainly in mRNA synthesis^19^. Rpb4/7 is thought to have an additional non-transcriptional role in transcription-coupled DNA repair mechanisms. The Rpb4/7 heterodimer can shuttle between nucleus and cytoplasm bound to mRNA and prevent it from degradation, implicating a role in mRNA export and translation. Furthermore, the RNA-binding protein Nrd1, involved in 3′ end formation of small nucleolar and nuclear RNAs during transcriptional termination, appears to interact with Rpb7^16,20,21^.

The cryo-EM analysis of the RNAP II/DSIF complex and X-ray crystallography revealed that the hSpt5 KOW4 requires a structural element at its N-terminus, KOWx-4, for stability^10^. Moreover, the C-terminal region of hSpt5 also includes a tandem KOW domain, designated KOW6-7, which is positioned near the exiting RNA, suggesting a function in recruiting factors for RNA capping and in 3′ RNA processing^22,23^.

To explore in depth the functions of KOW4 and KOW6-7, we determined the solution structures of KOW4 (S522-G647) and KOW6-7 (G961-A1087) of hSpt5 by solution nuclear magnetic resonance (NMR) spectroscopy and performed *in vitro* interaction studies with nucleic acids and Rpb4/7. Fluorescence and NMR-based titrations showed that only KOW4 variants bind to nucleic acids with micromolar to nanomolar dissociation constants and interact weakly with Rpb4/7. For KOW6-7 no binding to nucleic acids or Rpb4/7 could be detected.

## Results and Discussion

### Solution structures

**Figure 1:**
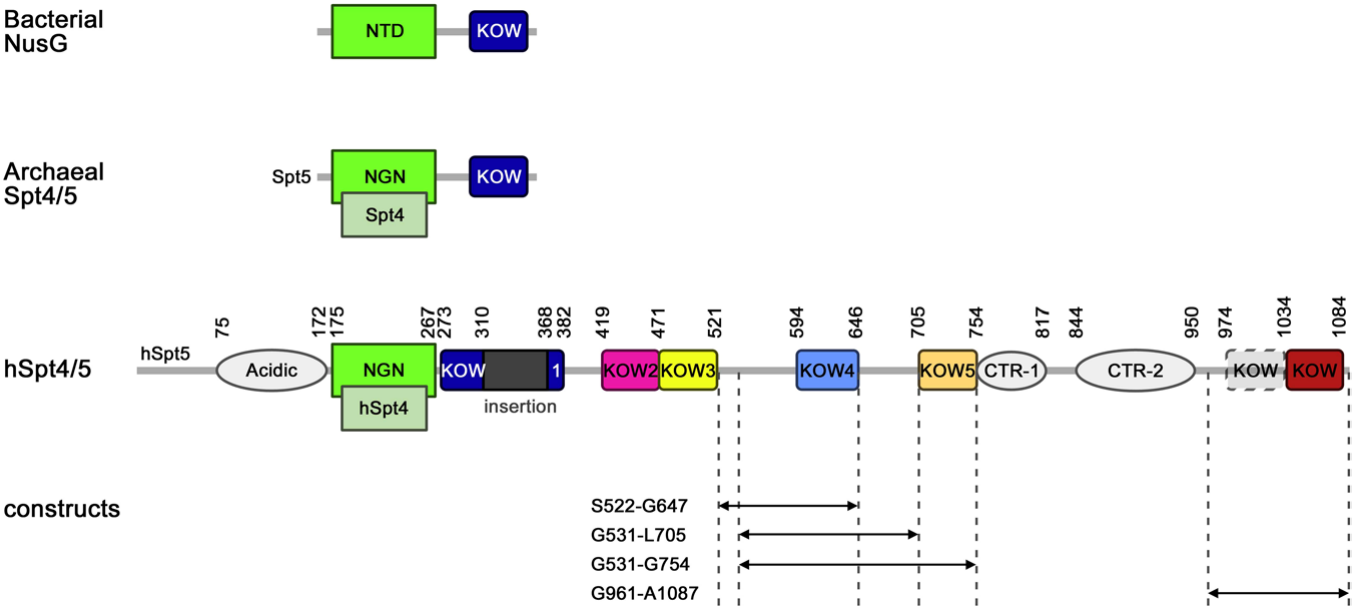
Schematic representation of Spt4 and Spt5 proteins. Bacterial NusG consists of an N-terminal domain (NTD) followed by one KOW domain. The archaeal dimeric Spt4/5 consists of a NusG N-terminal (NGN) domain in Spt5, which is homologous to NusG and interacts with Spt4, and one KOW domain. Eukaryotic Spt4/5 and the human DSIF (hSpt4/5) harbour an additional N-terminal acidic region that is followed by the NGN domain. KOW1-5 are found in all eukaryotes, however the CTRs and the C-terminal region adjacent to KOW5 are only present in the Spt5 of metazoans and plants. Numbers represent amino acid positions, dotted lines indicate the regions of the KOW4 and KOW6-7 variants used in this study.

During transcription elongation RNAP II associates with DSIF which harbours several KOW domains that have been implicated in different RNA and protein interactions. To probe the functions of KOW4 and KOW6-7, we constructed several variants comprising KOW4 or the C-terminal region of hSpt5 for solution structure determination and interaction studies (Fig. 1). We were able to determine the solution structure of a minimal KOW4 domain spanning the hSpt5 region from amino acid S522 to G647 using multidimensional NMR spectroscopy. N-terminal truncations harbouring only the region of the predicted KOW4 domain did not yield stable proteins (data not shown)^24^. In addition, we determined the solution structure of the C-terminal KOW6-7 using a construct spanning G961 to A1087 (Fig. 1 and Table 1).

**Table 1:** Solution structure statistics for KOW4 (S522-G647) and KOW6-7 (G961-A1087).

Experimentally derived restraints		KOW4	KOW6-7
**Distance restraints**			
	NOE	628	544
	intraresidual	17	14
	sequential	167	135
	medium range	82	85
	long range	362	310
	hydrogen bonds	2*31	2*46
interdomain NOEs		52	47
dihedral restraints		120	124
projection restraints (RDC)	^1^D (^1^H, ^15^N)		80
**Restraint violation**			
average distance restraint violation (Å)		0.0063 +/− 0.0007	0.0036 +/− 0.001
maximum distance restraint violation (Å)		<0.10	0.11
average dihedral restraint violation (°)		0.15 +/− 0.03	0.32 +/− 0.10
maximum dihedral restraint violation (°)		1.38	4.21
average RDC restraint violation (Hz)			0.21 +/− 0.03
Maximum RDC restraint violation (Hz)			1.16
**Deviation from ideal geometry**			
bond length (Å)		0.00062 +/− 0.00003	0.00042 +/− 0.00004
bond angle (°)		0.11 +/− 0.006	0.092 +/− 0.005
**Coordinate precision** ^a,b^			
backbone heavy atoms (Å) (all/defined structured region)		0.91/0.69	0.57
all heavy atoms (Å)		1.53/1.22	1.04
Ramachandran plot statistics^c^ (%)		88.3/11.5/0.3/0.0	88.8/10.7/0.5/0.0

^a^The precision of the coordinates is defined as the average atomic root mean square difference between the accepted simulated annealing structures and the corresponding mean structure calculated for the given sequence regions.

^b^Calculated for residues 536–646 (all) or 536–575, 597–646 (defined structured region) (KOW4) and 978–1085 (all, KOW6-7).

^c^Ramachandran plot statistics were determined by PROCHECK and noted by most favored/additionally allowed/generously allowed/disallowed.

**Figure 2:**
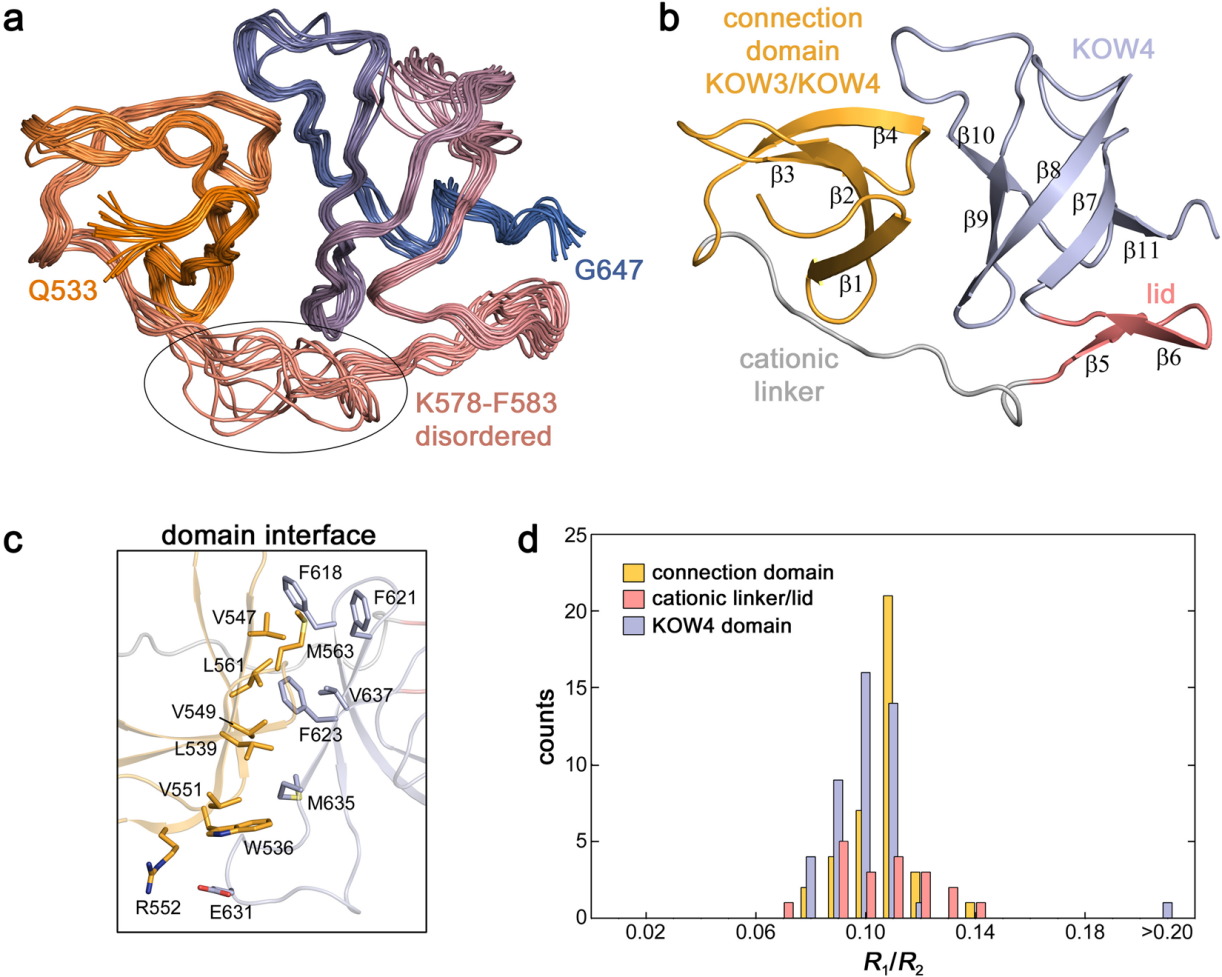
Solution structure of KOW4 (S522-G647). (**a**) Superposition of the 14 lowest energy structures. The disordered loop from K578 – F583 is encircled. The region from S522 to G532 is unstructured and not shown. (**b**) Ribbon representation depicting the region formerly described as a connection domain (β1–β4) in bright orange, the cationic linker in grey, the lid (β5–β6) in salmon, and the KOW4 domain (β7–β11) in light blue. (**c**) Stick representation showing relevant amino acids of the domain interface. (**d**) Distribution of *R*_1_/*R*_2_ for KOW4 (S522-G647).

The solution structure of KOW4 (S522-G647) shows the β-barrel fold typical for KOW domains (β-strands 7–11) that is covered by a β-hairpin (β-strands 5–6) called lid and stabilized N-terminally by a tilted, convex β-sheet (β-strands 1–4) (Fig. 2a,b). This N-terminal structural feature, formerly described as the connection domain between KOW3 and KOW4, is connected to the lid *via* a cationic linker and exhibits neither a typical KOW fold nor a KOW motif. It is composed mainly of a four-stranded antiparallel β-sheet (β-strands 1–4) with the strand order found in typical KOW domains. However, in KOW domains a fifth β-strand at the C-terminus adds to the β-sheet by pairing with the first strand, thereby forming the barrel-like domain. As the fifth β-strand is absent the region comprising only β-strands 1–4 should not be regarded as a typical KOW domain. It is required to stabilize the KOW4 domain and is connected to it by a cationic linker spanning K578 to F583.

The two domains interact at a highly apolar interface of about 700 Å^2^ in size. The interface mainly involves residues W536, L539, V547, V549, V551, M563 and L561 in the connection domain as well as F618, F621, F623, M635 and V637 in the KOW4 domain. In addition, the interface may be stabilized by a salt bridge between R552 and E631 (Fig. 2c).

To further characterize the domain interaction, we conducted ^15^N-based spin relaxation experiments. ^15^N relaxation rates could be determined for 117 residues (Fig. S1a). The heteronuclear {^1^H}^15^N steady state nuclear Overhauser effect (hetNOE) at 14 T shows values in the range of 0.7–0.8 for residues, except for the region N581-V585 and the termini. The hetNOE provides information about the motion of individual N-H bond vectors on the sub-ns timescale, significantly faster than the overall rotational tumbling. The values obtained are characteristic for restricted dynamics on the timescale of molecular tumbling for folded proteins. The hetNOE decreases towards the termini, demonstrating the increased flexibility on this timescale as is typical for unstructured termini. The cationic linker region shows slightly reduced values for the hetNOE indicating an enhanced flexibility on the sub-ns timescale. The transverse and longitudinal relaxation rates of ^15^N spins are very similar throughout the folded region of the protein, and the narrow distribution of the *R*_1_/*R*_2_ ratios suggests uniform overall tumbling of the protein (Fig. 2d). For proteins with flexibly connected domains, the relative domain motion is reflected in different distributions of the *R*_1_/*R*_2_ ratios of the two domains^25–27^. This is not the case for the KOW4 (S522-G647) construct. The fold of the KOW4 (S522-G647) domain deviates from the ideal sphere-like shape suggesting that the overall tumbling cannot be described by an isotropic rotation with a single correlation time. Determination of the rotational diffusion tensor based on the ^15^N relaxation rates requires a structural model and is a suitable method to validate the determined structure. The overall tumbling of KOW4 (S522-G647) can be well described by a prolate axial symmetric rotational diffusion tensor (Table 2) using 80 residues (36 residues for the connection domain and 44 for the KOW4 domain). A description by an isotropic rotation or an oblate axial symmetric tensor was rejected due to the χ^2^ statistics. The total asymmetric tensor did not significantly improve the result. The axial rotational diffusion tensor coincides well with the overall shape of the molecule (Fig. S1b) and the determined structure fits well with the relaxation data. Together with the numerous interdomain NOE spectroscopy (NOESY) cross-signals (52), these data confirm tight interdomain interaction.

**Table 2:** Rotational diffusion tensor analysis for KOW4 (S522-G647) (80 vectors).

isotropic		axialsymmetric (prolate)		axialsymmetric (oblate)		asymmetric	
		***D***_***⊥***_ **(10**^**8**^ **s**^**−1**^**)**^**a**^	0.155	***D***_***⊥***_ **(10**^**8**^ **s**^**−1**^**)**^**a**^	0.180	***D***_***x***_ **(10**^**8**^ **s**^**−1**^**)**	0.154
		***D***_***||***_ **(10**^**8**^ **s**^**−1**^**)**^**a**^	0.202	***D***_***||***_ **(10**^**8**^ **s**^**−1**^**)**^**a**^	0.158	***D***_***y***_ **(10**^**8**^ **s**^**−1**^**)**	0.156
						***D***_***z***_ **(10**^**8**^ **s**^**−1**^**)**	0.202
*t*_*c*_ (ns)	9.68 ± 0.04						
${\chi }_{\exp }^{{\rm{b}}}$	1.50 · 10^2^		8.44 · 10^1^		1.32 · 10^2^		8.43 · 10^1^
${\chi }_{0.1}^{{\rm{c}}}$	9.55 · 10^1^		9.22 · 10^1^		9.18 · 10^1^		8.96 · 10^1^
${\chi }_{0.05}^{{\rm{c}}}$	9.88 · 10^1^		9.81 · 10^1^		9.75 · 10^1^		9.52 · 10^1^

^a^*D*_*||*_ = *D*_*z*,_ *D*_*⊥*_ = *D*_*x*_ = *D*_*y*_ for the axialsymmetric model.

^b^*χ*^2^ = Σ (T_1i,exp_ − T_1i,calc)_^2^/σ(T_1i_) + Σ (T_2i,exp_ − T_2i,calc)_^2^/σ(T_2i_).

^c^Confidence limits (alpha = 0.1 or 0.05) of 500 Monte Carlo simulations. Models were accepted if *χ*_exp_ < *χ*_0.1_.

**Figure 3:**
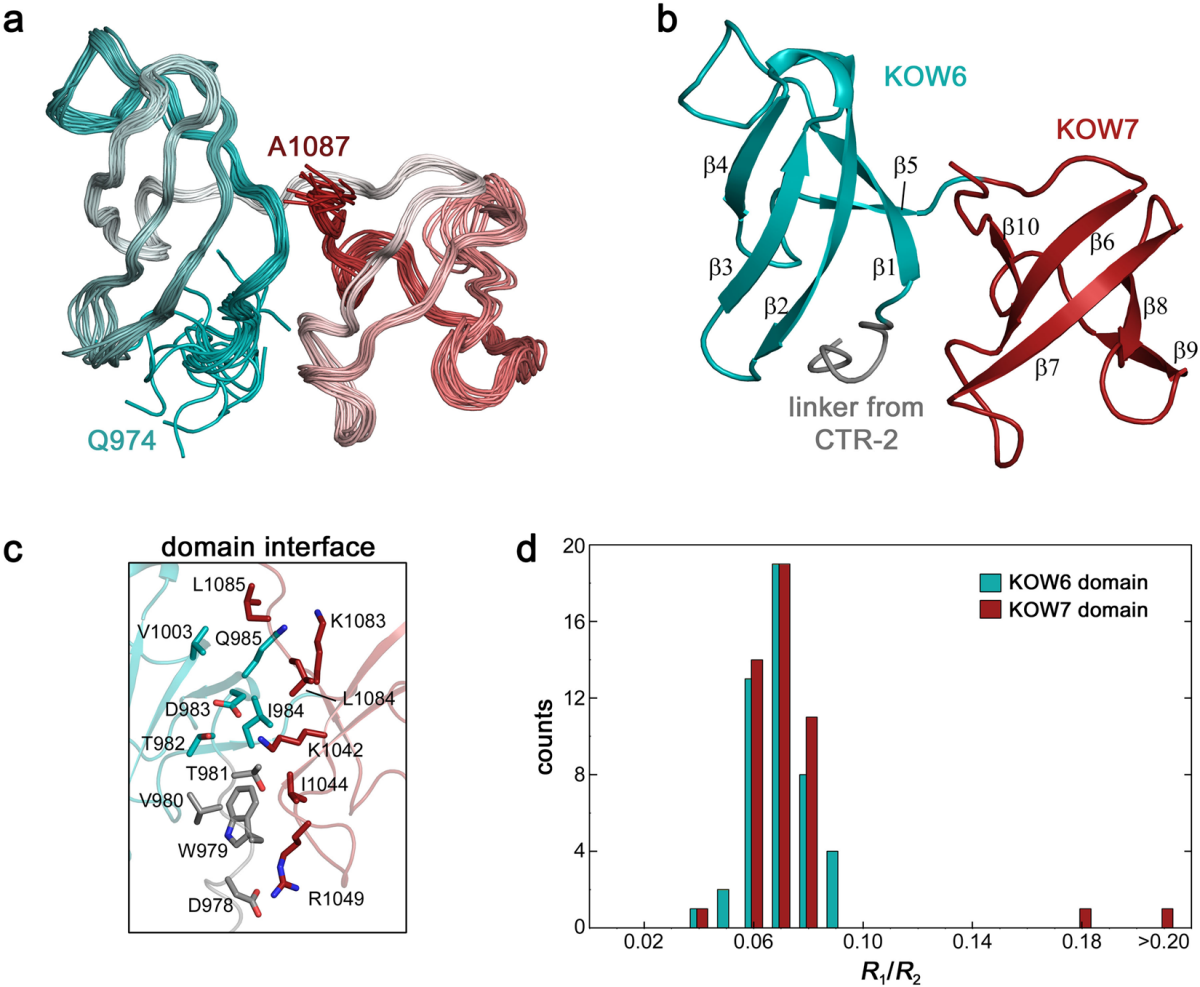
Solution structure of KOW6-7 (G961-A1087). (**a**) Superposition of the 20 lowest energy structures from Q974-A1087. The region from G961 to E973 is unstructured and not shown. (**b**) Ribbon representation highlighting KOW6 β1–β5) in cyan, and KOW7 (β6–β10) in red (**c**) Stick representation showing relevant amino acids of the domain interface. (**d**) Distribution of *R*_1_/*R*_2_ for KOW6-7 (G961-A1087). Colour assignment as in (b).

The C-terminal KOW harbouring region (G961-A1087) is also larger than predicted previously^7^. The solution structure reveals that it is composed of two domains, both sharing the typical KOW domain β-barrel fold (KOW6-7) (Fig. 3), is in good agreement with the crystal structure of a similar construct determined recently^10^. The linker between KOW6 and KOW7 consists of only five amino acids and the domain interface of about 500 Å^2^ is characterized by a small number of hydrophobic residues in the centre (W979, I984 and I1044) that are surrounded by polar residues. In particular, one lysine residue and one arginine residue (K1042, R1049) in KOW7 may form salt bridges with aspartates (D978 and D983) from the linker/KOW6 side (Fig. 3c). This makes this domain interaction distinct from that in KOW4 (S522-G647), which almost exclusively consists of hydrophobic interface residues.

As described above for KOW4 (S522-G647) we performed ^15^N-based spin relaxation experiments to characterize the domain movement of KOW6-7 (G961-A1087). ^15^N relaxation rates were obtained for 112 non-overlapping amide resonances. The hetNOE experiments show the typical values around 0.7–0.8 for the folded part, demonstrating the highly restricted flexibility for the N-H bond vectors of this region. The flexibility on the sub-ns timescale increases towards the termini. For the region linking both domains no increased dynamical behaviour on the sub-ns timescale is observed. Similarly to KOW4 (S522-G647), the narrow, monomodal distribution of *R*_1_*/R*_2_ ratios suggests that the two KOW domains of KOW6-7 (G961-A1087) interact and the protein moves as one entity (Fig. 3d). Analysis of the rotational diffusion tensor using the ^15^N relaxation rates (Fig. S2a) of 79 residues (37 residues for KOW6 and 42 for KOW7) and the determined structure show that the overall tumbling can be well described by a prolate axial symmetric tensor (Table 3 and Fig. S2b). Similar to the results obtained with KOW4 (S522-G647), employment of the total asymmetric tensor did not improve the fit, and the isotropic rotation and the oblate axial symmetric tensor were rejected due to the χ^2^ statistics. Together with 47 interdomain NOE cross signals, the overall tumbling as single entity demonstrates the tight domain interaction of KOW6 and KOW7.

**Table 3:** Rotational diffusion tensor analysis for KOW6-7 (G981-A1087) (79 vectors).

isotropic		axialsymmetric (prolate)		axialsymmetric (oblate)		asymmetric	
		***D***_***⊥***_ **(10**^**8**^ **s**^**−1**^**)**^**a**^	0.145	***D***_***⊥***_ **(10**^**8**^ **s**^**−1**^**)**^**a**^	0.171	***D***_***x***_ **(10**^**8**^ **s**^**−1**^**)**	0.143
		***D***_***||***_ **(10**^**8**^ **s**^**−1**^**)**^**a**^	0.193	***D***_***||***_ **(10**^**8**^ **s**^**−1**^**)**^**a**^	0.138	***D***_***y***_ **(10**^**8**^ **s**^**−1**^**)**	0.148
						***D***_***z***_ **(10**^**8**^ **s**^**−1**^**)**	0.192
*t*_*c*_ (ns)	10.36 ± 0.04						
${\chi }_{\exp }^{{\rm{b}}}$	1.96 · 10^2^		8.11 · 10^1^		1.29 · 10^2^		8.06 · 10^1^
${\chi }_{0.1}^{{\rm{c}}}$	9.56 · 10^1^		8.91 · 10^1^		9.12 · 10^1^		8.76 · 10^1^
${\chi }_{0.05}^{{\rm{c}}}$	9.99 · 10^1^		9.31 · 10^1^		9.42 · 10^1^		9.11 · 10^1^

^a^*D*_*||*_ = *D*_*z*_, *D*_*⊥*_ = *D*_*x*_ = *D*_*y*_ for the axialsymmetric model.

^b^*χ*^2^ = Σ(T_1i,exp_ − T_1i,calc)_^2^/σ(T_1i_) + Σ(T_2i,exp_ − T_2i,calc)_^2^/σ(T_2i_).

^c^Confidence limits (alpha = 0.1 or 0.05) of 500 Monte Carlo simulations. Models were accepted if *χ*_exp_ < *χ*_0.1_.

**Figure 4:**
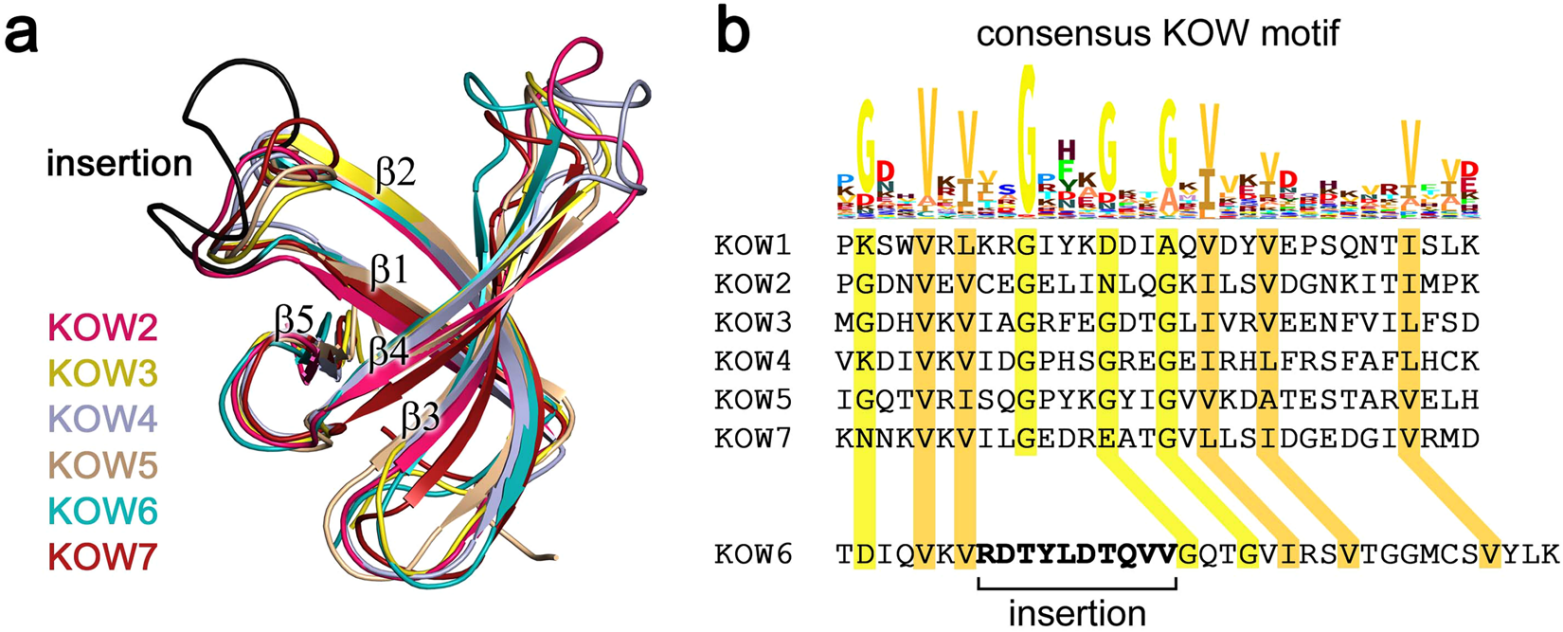
Structural overlay of hSpt5 KOW domains and sequence comparison with the consensus KOW motif. (**a**) The structures of hSpt5 KOW2 (PDB: 2E6Z), KOW3 (PDB: 2DO3), KOW4 (S522-G647) (this work, PDB: 6EQY), KOW5 (PDB: 2E70), and KOW6-7 (G961-A1087) (this work, PDB: 6ER0) were used for the overlay. (**b**) Comparison of the hSpt5 KOW sequences with the consensus KOW motif highlights the conserved residues in yellow and orange and the insertion in KOW6 in bold.

An overlay of all available hSpt5 KOW domain structures discloses that they all share the typical KOW domain β-barrel fold (Fig. 4a). The first of the two KOW domains of KOW6-7 (G961-A1087) (β-strands 1–5), designated as KOW6, had not been identified previously by sequence analysis. KOW6 lacks one highly conserved glycine residue characteristic for a KOW motif. Instead, an insertion between β-strands 1 and 2 results in a larger loop of unknown function between these two β-strands (Fig. 4a,b).

In summary, both KOW4 (S522-G647) and KOW6-7 (G961-A1087) exhibit intramolecular domain-domain interactions and enlarge the structure of the basic KOW fold compared to other KOW domains of hSpt5. The extended structure might be important to present a larger binding surface for additional molecular interactions.

### Substrate binding

**Figure 5:**
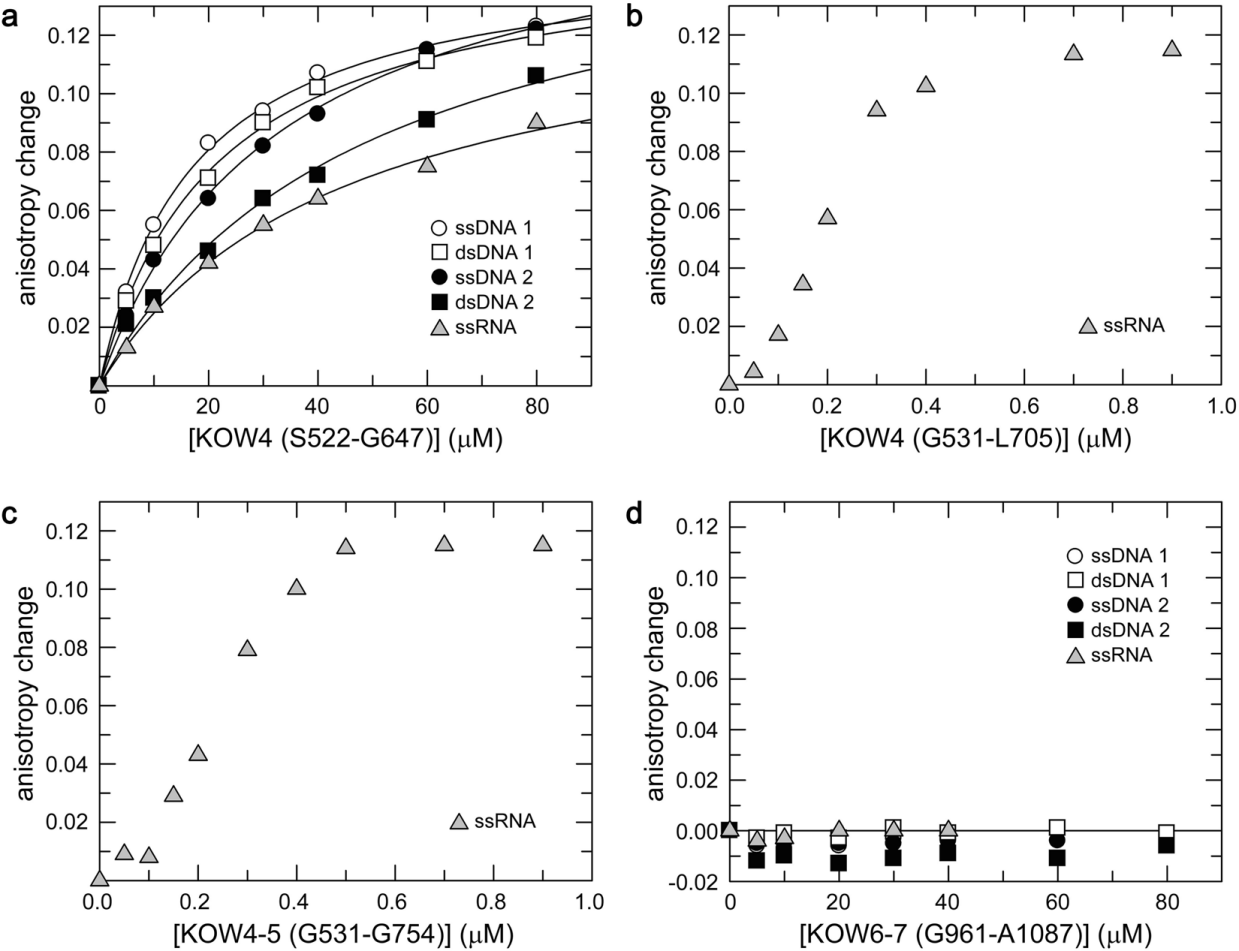
Determination of nucleic acid binding affinities by fluorescence anisotropy measurements. 50 nM (**a**,**d**) or 25 nM (**b**,**c**) of fluorescent labelled nucleic acids as indicated were titrated with (**a**) KOW4 (S522-G647), (**b**) KOW4 (G531-L705), (**c**) KOW4 (G531-G754), (**d**) KOW6-7 (G961-A1087). The curves in (**a**) represent the best fit to a two-component binding equation to determine the *K*_D_ values^31^ for ssDNA1 (12.2 ± 0.6 µM), dsDNA1 (20.1 ± 0.4 µM), ssDNA2 (24.5 ± 3.2 µM), dsDNA2 (41.2 ± 9.0 μM), and ssRNA (39.5 ± 7.4 μM).

The cryo-EM structure of the RNAP II/DSIF complex and the crystal structure of the complex from yeast indicated that the linker region between KOW4 and KOW5 is part of the RNA clamp that guides exiting RNA^10,28^. Thus, we investigated the affinity of several KOW4-5 variants (Fig. 1) and of KOW6-7 (G961-A1087) for nucleic acid substrates. Determination of the *K*_*D*_ values by fluorescence anisotropy titrations indicated that KOW4 (S522-G647) has similar sequence-independent micromolar range affinities for DNA and RNA, with some preference for single-stranded substrates (Fig. 5a).

We also used KOW4 variants that included the KOW4-KOW5 linker, KOW4 (G531-L705) (Fig. 5b), or, in addition, the KOW5 domain, KOW4-5 (G531-G754) (Fig. 5c), and titrated an ssRNA substrate. Since the data points obtained with both constructs exhibited sigmoidal binding kinetics, implying a complex binding behaviour, we were not able to determine *K*_*D*_ values using a two-state binding model. Nevertheless, the data for both constructs indicated similar binding affinities for RNA in the high nanomolar range. We have not defined the mode of RNA binding, however. Compared to KOW4 (G531-L705), the presence of KOW5 in the construct KOW4-5 (G531-G754) did not further enhance RNA binding. These data suggest a substantial contribution of the linker region G648-G705 between KOW4 and KOW5, but not of KOW5 itself, to RNA binding (Fig. 5b,c). These results are in good agreement with the structural data of the RNAP II/DSIF complex, which showed that the KOW4-KOW5 linker is part of the RNA clamp and that KOW 5 primarily interacts with the Rpb1 dock as well as the Rpb2 wall domains to enhance transcription elongation^10,28^.

Although the twin KOW6-7 domains are positioned near the exiting RNA in the cryo-EM structure of the RNAP II/DSIF complex^10^, we could not detect nucleic acid binding for the corresponding KOW6-7 (G961-A1087) construct, indicating that it probably interacts exclusively with protein factors necessary for RNA elongation and/or RNA processing during transcription termination (Fig. 5d).

### Determination of the nucleic acid binding site of KOW4 by NMR

**Figure 6:**
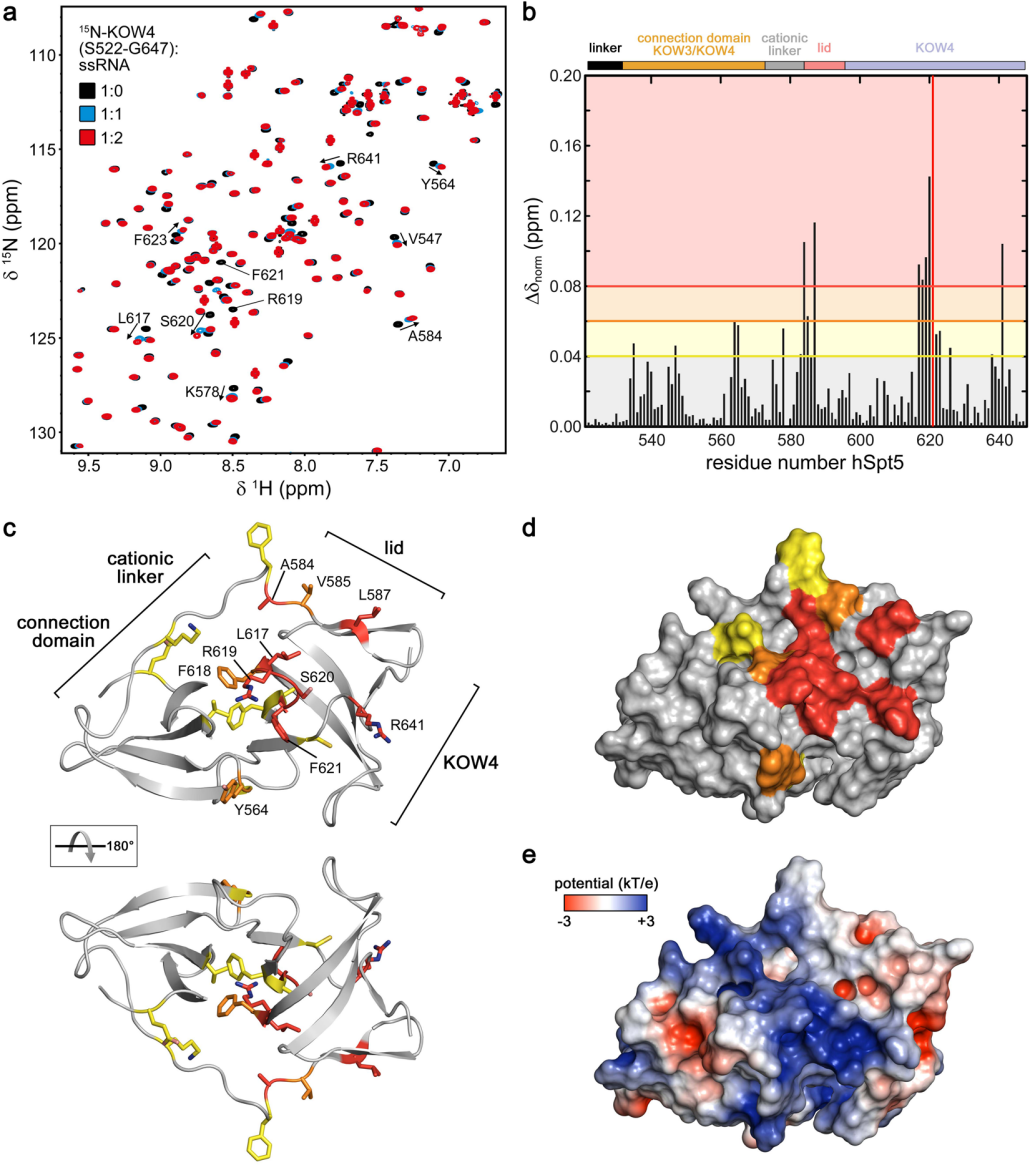
Determination of the KOW4 (S522-G647) nucleic acid binding interface. (**a**) Overlay of [^1^H, ^15^N] HSQC spectra recorded during titration with different protein (70 µM):ssRNA ratios as indicated. Relevant residues affected by ssRNA addition are labelled by arrows. (**b**) Normalized chemical shift changes upon ssRNA binding. Changes larger than 0.04 ppm were considered significant, changes from 0.04 to 0.06 ppm were assigned as weak, >0.06–0.08 ppm as medium, and >0.08 ppm as strong. The different regions of KOW4 (S522-G647) are indicated on top of the diagram. (**c**,**d**) Mapping of the observed chemical shift changes colour coded as in (**b**) on the structure of KOW4 (S522-G647) in ribbon (**c**) and surface representation (**d**). The amino acids exhibiting significant chemical shift changes are indicated as yellow (weak), orange (medium) and red (strong). (**e**) Electrostatic surface potential of KOW4 (S522-G647) calculated with the program APBS^42^, coloured from −3 kT/e to +3 kT/e.

To determine the nucleic acid binding site on KOW4, we conducted 2D [^1^H, ^15^N] heteronuclear single quantum correlation (HSQC)-based titration experiments of ^15^N-labelled KOW4 (S522-G647) with ssRNA (Fig. 6a), ssDNA and dsDNA (Fig. S3a,b). In each titration experiment, we observed chemical shift changes for some signals, indicating fast chemical exchange on the NMR time scale. Analysis of the chemical shift perturbations revealed the strongest effects on amino acids in the cationic linker and the lid region (around position 585) as well as in the centre of KOW4 (S522-G647) (around position 620) (Figs 6b and S3c,d).

Most residues affected by nucleic acid binding form a well-connected patch centred at the loop between the second and third β-strand of KOW4 (S522-G647). Moreover, most binding site residues carry a polar, positively charged, or aromatic side chain (Fig. 6c,d). This is consistent with their ability to contact the phosphate backbone and bases of the nucleic acid binding partner. KOW4 (S522-G647) includes a positively-charged patch formed by the KOW4 β-barrel and the cationic linker that matches the position of the nucleic acid-binding site (Fig. 6e). The position of the RNA binding region determined here supports the structural data of the RNAP II/DSIF complex in which the KOW4 β-barrel contacts the exiting RNA as part of the RNA clamp^10^.

Since the fluorescence anisotropy measurements revealed that the binding affinities for nucleic acids of KOW4 (G531-L705), which harbours the KOW4-KOW5 linker region, were higher than of KOW4 (S522-G647), we used an overlay of the 2D [^1^H, ^15^N] HSQC spectra of the two ^15^N labelled proteins to identify the signals corresponding to the KOW4-KOW5 linker (Fig. S4). The spectrum of KOW4 (G531-L705) demonstrates that the linker signals are located in the random coil region and that not all 58 residues of the linker are visible. This is probably due to fast exchange with the solvent and/or line broadening caused by conformational exchange. Titration of KOW4 (G531-L705) with ssRNA did not result in additional chemical shift perturbations in the linker region. Possibly, several linker region arginine residues contribute to binding without forming a defined structure. Indeed, mutagenesis studies suggested that these residues participate in RNA binding and in overall stabilization of the elongation complex^10^.

These results in combination with the fluorescence titration experiments described above indicate a role of the linker region A584-G705 in RNA binding.

### Binding of the RNAP II subunit complex Rpb4/7 to KOW4 S522-G647

**Figure 7:**
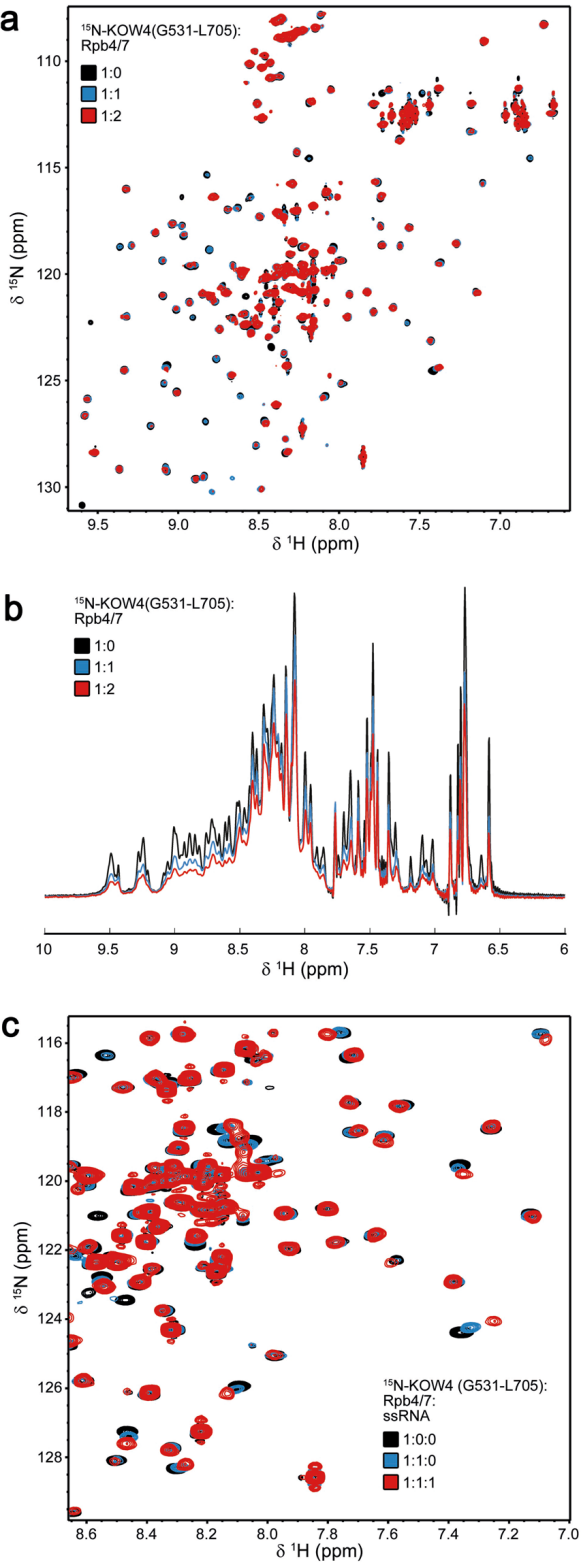
Binding of KOW4 (G531-L705) to Rpb 4/7. Proteins were measured in a buffer containing 50 mM sodium phosphate, pH 7.0, 50 mM NaCl and 1 mM DTT at 298 K. (**a**) 2D [^1^H, ^15^N] HSQC and (**b**) 1D [^1^H, ^15^N] HSQC spectra of 150 µM ^15^N labelled KOW4 (G531-L705) before (black), and after the addition of Rpb4/7 (molar ratio 1:1, 50 μM each, blue; molar ratio 1:2, KOW4 (G531-L705) 41 μM, red) (**c**) 2D [^1^H, ^15^N] HSQC spectra of 50 μM ^15^N-labelled KOW4 (G531-L705) in the absence (black) or presence (blue) of equimolar concentrations of Rpb4/7, and after the addition of ssRNA (molar ratio 1:1:1, 45 μM each, red).

*In vivo* cross-linking experiments suggested an interaction of KOW4 and of the linker between KOW4 and KOW5 with the Rpb4 and Rpb7 subunits of RNAP II^16^. The 3D structures of the yeast and human RNAP II/DSIF complexes confirmed contacts between the Rpb4/7 stalk and KOW4. However, the precise location and orientation of KOW4 differs in the yeast and human RNAP II/DSIF complexes^10,28^. To analyse the interaction between Rpb4/7 and the KOW4 domain including the KOW4-KOW5 linker, we expressed and purified the human Rpb4/7 heterodimer and performed *in vitro* NMR titration experiments with ^15^N labelled KOW4 (G531-L705) (Fig. 7). The 2D [^1^H, ^15^N] HSQC spectra indicate weak interaction of Rpb4/7 with KOW4 (G531-L705) since only small chemical shift changes could be detected for the 1:1 complex (Fig. 7a). However, the 1D [^1^H, ^15^N] HSQC spectra of the titration confirmed the interaction of Rpb4/7 with KOW4 (G531-L705) (Fig. 7b). Signals of ^15^N labelled KOW4 (G531-L705) (19.3 kDa) decreased significantly upon addition of unlabelled Rpb4/7 (36.6 kDa), indicating complex formation (Fig. 7b). The increase in molecular mass upon binding results in faster magnetisation relaxation and thus line broadening.

Together with the titration experiments of KOW4 (S522-G647) with RNA (Fig. 6) these results support the notion of interactions between KOW4 and Rpb4/7 and/or RNA.

Comparison of the [^1^H, ^15^N] HSQC spectra of KOW4 titrated with either Rpb4/7 (Fig. 7a) or RNA (Fig. 6) demonstrated that several of the affected residues were identical, suggesting similar or overlapping binding sites for Rpb4/7 and ssRNA. To confirm this, we carried out a HSQC-based displacement experiment (Fig. 7c). We added ssRNA to the preformed ^15^N-KOW (G531-L705)/Rpb4/7 complex. The [^1^H, ^15^N] HSQC spectrum shows chemical shift changes of the same signals that were already affected upon addition of Rpb4/7 to KOW4 (G531-L705) (Fig. 7c). No additional chemical shift changes could be observed, indicating that ssRNA can displace Rpb4/7 and that the affinity of ssRNA to KOW4 (G531-L705) is higher than that of Rpb4/7.

## Conclusion

We postulate that in a transcription initiation complex, the KOW4 domain of DSIF is loosely associated with the Rpb4/7 stalk of RNAP II. However, during elongation, when the transcribed RNA leaves the exit channel, the KOW4 domain and the KOW4-5 linker associate with the exiting RNA, thus stabilizing the elongation complex. Once the transcript is finished, Spt5 no longer binds to Rpb4/7. Without this stabilization, Rpb4/7 is able to dissociate from RNAP II and leave the nucleus together with the RNA, preventing its degradation. Thus Spt5, like NusG, could play a role in coupling transcription with translation, at least indirectly.

The structure of the elongating RNAP II/DSIF complex revealed that KOW4-5 forms an RNA clamp^10^. Comparison of the RNA binding affinities of KOW4 (S522-G647) lacking the linker and KOW4 (G531-L705) which includes the linker, further indicated that the linker between KOW4 and 5 contributes substantially to RNA binding (Fig. 5).

Interestingly, KOW6-7 (G961-A1087) exhibits a similar spatial arrangement as has been determined for the CTD of human KIN17, which harbours two SH3-like domains (PDB: 2CKK). KIN17 is a 45 kDa DNA and RNA binding protein that plays an important role in nuclear metabolism^29^. Similar to KOW6-7 (G961-A1087), the dimer interface of KIN17 harbours an Arg and a Lys residue (R351, K391). In addition, the protein also comprises an extended loop between β-strands 1 and 2. In contrast to hSpt5 KOW6-7 (G961-A1087), human KIN17 binds RNA. The negatively charged groove of the domain interface of KIN17 might constitute an additional surface for interaction with other proteins.

In the cryo-EM structure of the RNAP II/DSIF complex, no density was observed beyond the KOW5 domain^10^. Thus, no function for KOW6-7 could be determined. Since we find no direct interaction between KOW6-7 and nucleic acids, we propose that KOW6-7 might recruit other factors, for example proteins that play a role in RNA elongation, termination and processing, possibly *via* the positively charged amino acids flanking the groove (residues R1079, R989, K987, K1083, K1017) and the hydrophobic amino acids located within (W979, P1017, I1018).

## Materials and Methods

### Cloning, expression and protein purifications

#### KOW domains

The genes coding for KOW4 variants (S522-G647), (G531-L705) and (G531-G754) and KOW6-7 (G961-A1087) of hSpt5 were amplified by PCR using cDNA plasmid pOTB7 huSUPT5H (open biosystems, GE Healthcare) as a template. 5′ and 3′ primers harbouring NcoI and BamH I restriction sites, respectively were used to clone the PCR fragments into the vector pET-GB1a (G. Stier, EMBL, Heidelberg, Germany). The proteins expressed were thus fused to the C-terminus of the B1 domain of streptococcal protein G (GB1) and could be released *via* a tobacco etch virus (TEV) protease cleavage site located between GB1 and the KOW domain. Gene expression in lysogeny broth or in M9 medium for ^15^N and ^13^C labelling was performed in *Escherichia coli* strain BL21 (DE3) (Invitrogen-Life Technologies, Darmstadt, Germany) as described^30^. After induction with 100 µM isopropyl-thiogalactoside (IPTG) the temperature was reduced to 20 °C and protein overexpression was performed overnight. Proteins were purified via Ni-affinity chromatography (HisTrap, GE Healthcare, Munich, Germany) and TEV cleavage followed by a second Ni-affinity chromatography which allowed the removal of the 6xHis-GB1 tag. The free KOW proteins were collected in the flow-through and purified further *via* anion exchange chromatography using a QXL column (GE Healthcare, Munich, Germany). All constructs were flash-frozen with liquid nitrogen and stored at −80 °C.

#### human Rpb4/7

Genes adapted for *E*. *coli* were cloned in tandem into the expression vector pET15b. Rpb4 harboured a sequence coding for an N-terminal 6His tag. *E*. *coli* BL21 (DE3) cells (Invitrogen-Life Technologies, Darmstadt, Germany) transformed with the expression plasmid were grown to an optical density at 600 nm of 0.7–0.9 at 37 °C in LB or M9 medium containing 100 µg/ml ampicillin. Gene expression was induced with 1 mM IPTG for 4 h at 37 °C. The cells were then harvested by centrifugation. Cell lysis was performed as described for the KOW constructs in 50 mM sodium phosphate (pH 6.8), 500 mM NaCl, 1 mM dithiothreitol (DTT). The heterodimer was purified *via* Ni-affinity chromatography (HisTrap, GE Healthcare, Munich, Germany), followed by anion exchange chromatography (5 ml QXL column, GE Healthcare, Munich, Germany) after dialysis against 20 mM Tris/HCl pH 6.8, 20 mM NaCl, 1 mM DTT). The protein complex was eluted with an NaCl step gradient. Rpb4/7 containing fractions were combined, dialyzed against 20 mM Tris/HCl pH 6.8, 20 mM NaCl, 1 mM DTT, concentrated by ultrafiltration, and stored at −80 °C after flash-freezing with liquid nitrogen.

### Fluorescence anisotropy measurements

Fluorescence anisotropy measurements were performed at 25 °C on a Synergy 2 microplate reader (biotek) equipped with black, sterile 96-well microtiter plates. The single stranded (ss) or double stranded (ds) DNAs or ssRNA were labelled with 6-FAM at the 5′ ends and contained the following sequences: ssDNA1: 6FAM-CTTATTGAATTA; ssDNA2: 6FAM-GAAAATTGGGTAAG; ssRNA: 6FAM-GGCGGUAGCGUG (metabion, Planegg, Germany). For dsDNA, the corresponding complementary strands without label were hybridized to ssDNA1 and 2 in fluorescence buffer (25 mM Tris/HCl pH 7.0, 50 mM NaCl) at a molar ratio of 1:1.2 (labelled:unlabelled strand) by heating the sample for 3 min at 95 °C, followed by a 10 min incubation step at 34 °C, or 40 °C for dsDNA1, or dsDNA2, respectively. Titrations were performed with individual samples, each containing 25 nM (for KOW4 (G531-L705), and KOW4 (G531-G754)) or 50 nM for KOW4 (G522-G647) and KOW6-7 (G961-A1087) of nucleic acid substrate and increasing amounts of protein in a total volume of 100 μl. The anisotropy of each sample was measured 6 times. Data were analysed by plotting the anisotropy value corrected for the value of the free nucleic acid vs. the protein concentration in the sample. The curves in Fig. 4a represent the best fit to a two-component binding equation describing the binding equilibrium to determine the *K*_D_ values^31^. All binding curves were measured in triplicates.

### NMR spectroscopy

All NMR experiments were conducted on Bruker Avance 600 MHz, 700 MHz, 900 MHz and 1000 MHz spectrometers, the latter three equipped with cryogenically cooled probes. Standard double and triple resonance experiments^32,33^ were conducted for backbone and sidechain resonance assignments at 298 K. ^15^N- and ^13^C-edited 3D NOESY experiments were recorded with mixing times of 120 ms at 298 K, in a buffer containing 20 mM sodium phosphate, pH 6.4, 20 mM NaCl, and 0.5 mM DTT.

^1^H, ^15^N residual dipolar couplings (RDCs) were determined for KOW6-7 (G961-A1087) by in-phase/anti-phase (IPAP) experiments^34^ using a sample containing 10 mg/ml Pf1 phages (AslaBiotech AB, Latvia)^35^. Determination of RDCs for KOW4 (S522-G647) were not successful using either Pf1 phages or mixtures of hexa-ethylene glycol monododecyl ether (C6E12), hexanol and water^36^ due to sample instability.

For characterization of overall and internal motions, ^15^N longitudinal (*R*_1_) and transverse (*R*_2_) relaxation rates together with the {^1^H}^15^N steady state NOE were determined using standard methods^37^ at 600.2 MHz (KOW4 (S522-G647)) or 700.2 MHz (KOW6-7 (G961-A1087)) ^1^H frequency at a calibrated temperature of 298 K.

*R*_1_ and *R*_2_ relaxation rates were determined by fitting a mono-exponential curve to the signal intensities using the CURVEFIT program (A.G. Palmer, Columbia University, USA). Rotational diffusion tensor analysis was done using the program tensor2^38^. The error of relaxation rates was set to 5% to reflect potential systematic errors due to pulse imperfections, different temperatures in *R*_1_ and *R*_2_ experiments due to different radio frequency heating, and potential structural noise in the structural models. Residues with {^1^H},^15^N steady state values below 0.7 and residues with enhanced *R*_2_ rates due to chemical exchange were not included in the analysis. The different models for the rotational diffusion tensor were accepted or rejected based on the χ^2^ statistics using 500 Monte Carlo simulations.

### Solution structure calculation

Distance restraints for structure calculation were derived from ^15^N-edited NOESY and ^13^C-edited NOESY spectra. NOESY cross peaks were classified according to their relative intensities and converted to distance restraints with upper limits of 3.0 Å (strong), 4.0 Å (medium), 5.0 Å (weak), and 6.0 Å (very weak). For ambiguous distance restraints the r^−6^ summation over all assigned possibilities defined the upper limit. Hydrogen bonds were included for backbone amide protons in regular secondary structure, when the amide proton did not show a water exchange cross peak in the ^15^N-edited NOESY spectrum.

Structure calculations were performed with the program XPLOR-NIH 1.2.1^39^ using a three-step simulated annealing protocol with floating assignment of prochiral groups including a conformational database potential. The 14 (KOW4) and 20 (KOW6-7) structures showing the lowest values of the target function excluding the database potential were further analysed with X-PLOR^39^, PyMOL, and PROCHECK 3.5.4^40^.

### Structure and sequence alignments

Structure alignments were performed using WinCoot’s secondary structure matching algorithm. Sequence alignment of the hSpt5 KOW motifs was performed manually, based on the KOW consensus sequence provided by Pfam^41^.

### Data deposition

The structure coordinates of hSpt5 KOW4 (S522-G647) and KOW6-7 (G961-A1087) were deposited in the Protein Data Bank under the accession codes 6EQY and 6ER0, respectively. Chemical shift assignments were deposited in the BioMagResBank accession numbers 34184 and 34185, respectively.

## Electronic supplementary material

Supplementary Information
