# Supplementary material for: Structure and nucleic acid binding properties of KOW domains 4 and 6–7 of human transcription elongation factor DSIF
Source: Sci Rep. 2018 Aug 3;8:11660. doi: 10.1038/s41598-018-30042-3 (PMC6076269; doi:10.1038/s41598-018-30042-3)
Supplement: Supplementary file 1 — Supplementary Information [file 41598_2018_30042_MOESM1_ESM.docx]

# Supplementary Information

**Structure and nucleic acid binding properties of KOW domains**

**4 and 6-7 of human transcription elongation factor DSIF**

**Philipp K. Zuber^1^, Lukas Hahn^1^, Anne Reinl^1^, Kristian Schweimer^1^, Stefan H. Knauer^1*^, Max E. Gottesman^3^, Paul Rösch^1,2^ and Birgitta M. Wöhrl^1*^**

^1^Universität Bayreuth, Lehrstuhl Biopolymere and ^2^Forschungszentrum für Bio-Makromoleküle, Universitätsstr. 30, D-95447 Bayreuth, Germany

^3^Department of Microbiology and Immunology, Columbia University, New York, NY USA.

*corresponding authors

#
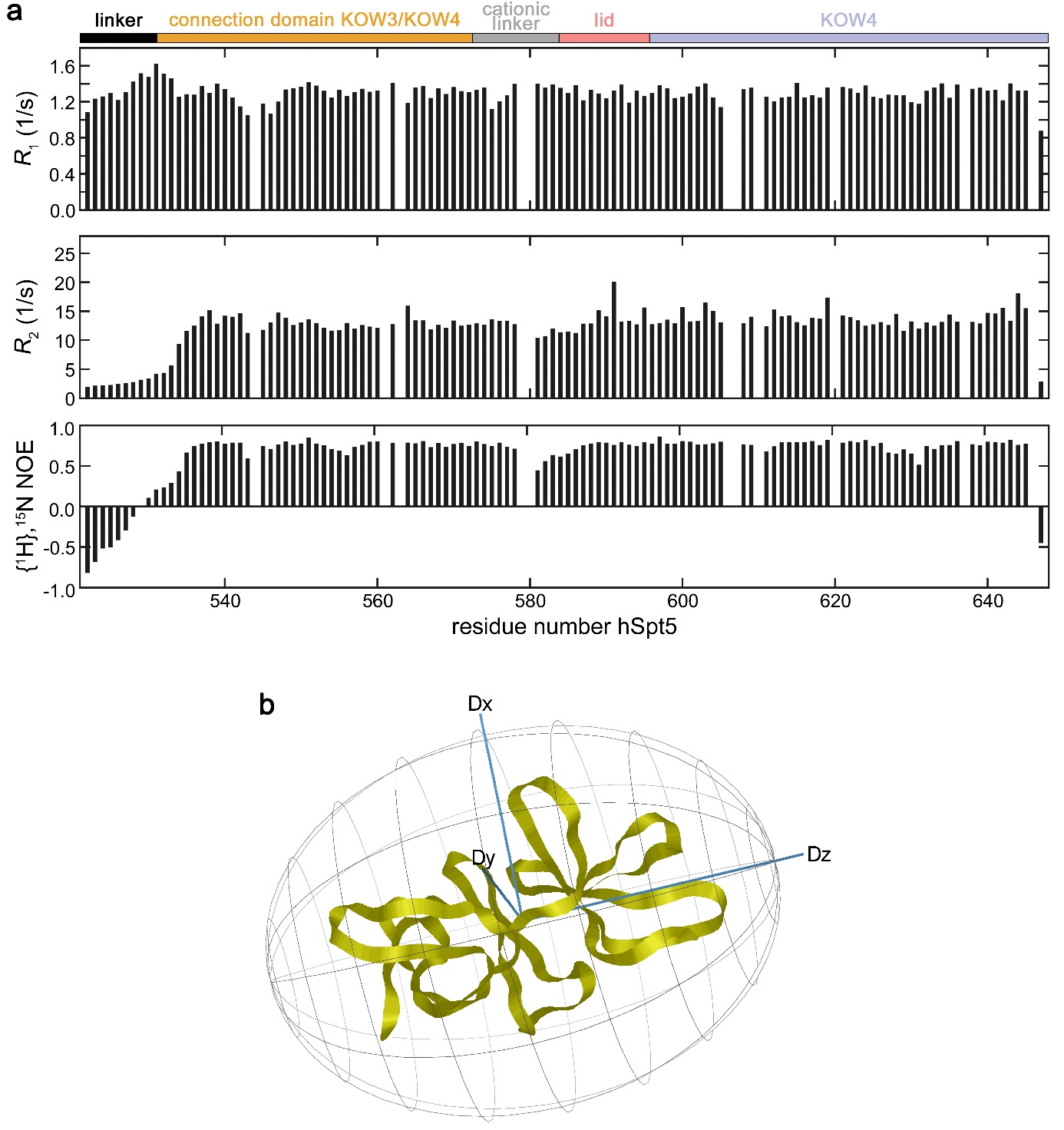


**Figure S1: ^15^N relaxation data and diffusion tensor analysis of KOW4 (S522-G647). (a)** Longitudinal (*R*_1_, top), transverse (*R*_2_, middle), and steady-state heteronuclear {^1^H} ^15^N NOE as a function of the KOW4 sequence position (S522-G647). **(b)** Diffusion tensor analysis. KOW4 (S522-G647) is shown as a yellow ribbon surrounded by the determined diffusion tensor (grey net).

**
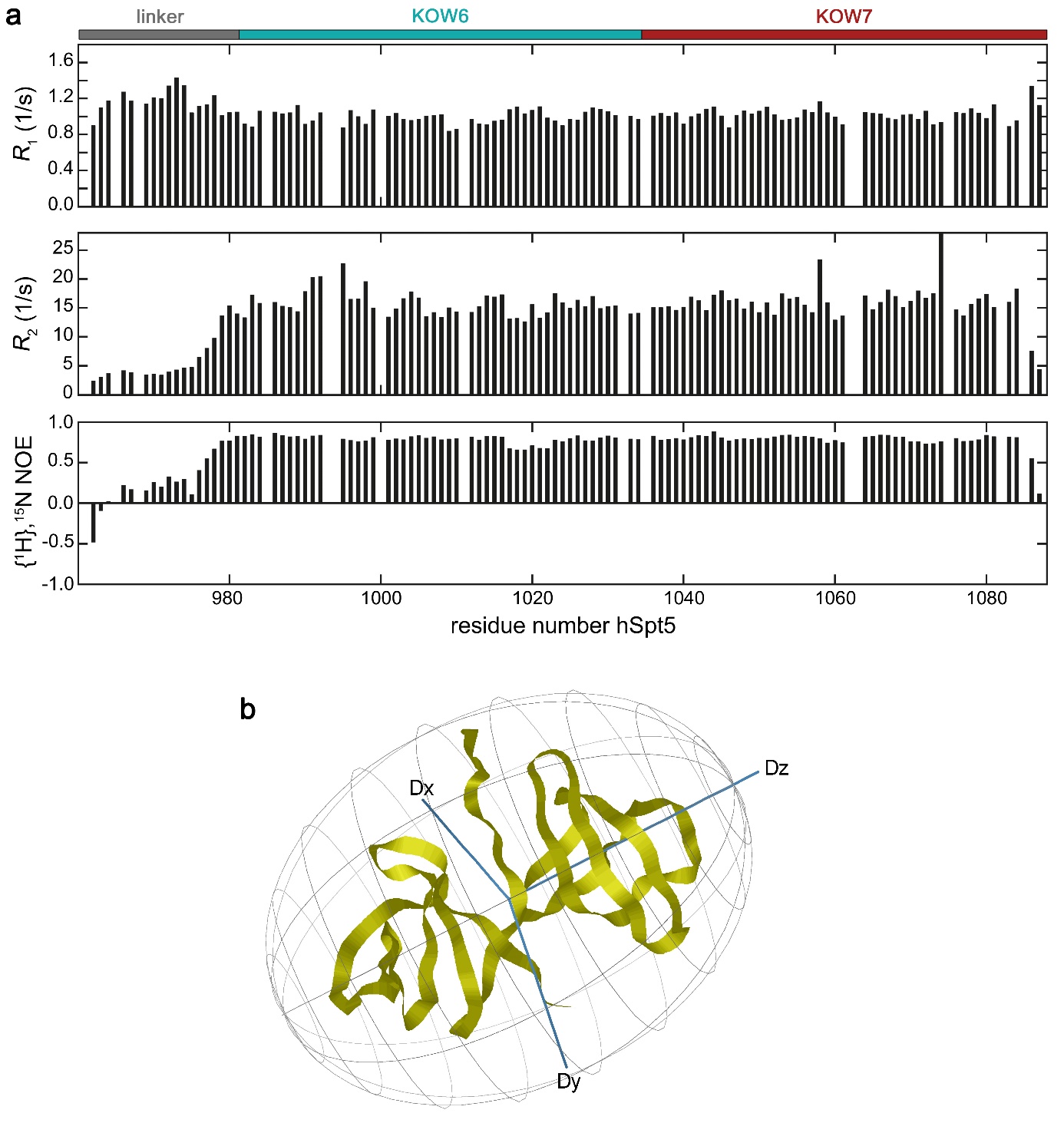
**

**Figure S2: ^15^N relaxation data and diffusion tensor analysis of KOW6-7 (G961-A1087). (a)** Longitudinal (R_1_, top), transverse (R_2_, middle), and steady-state heteronuclear {^1^H} ^15^N NOE as a function of sequence position of KOW6-7 (G961-A1087). **(b)** Diffusion tensor analysis. KOW6-7 (G961-A1087) is shown as a yellow ribbon surrounded by the determined diffusion tensor (grey net).

**
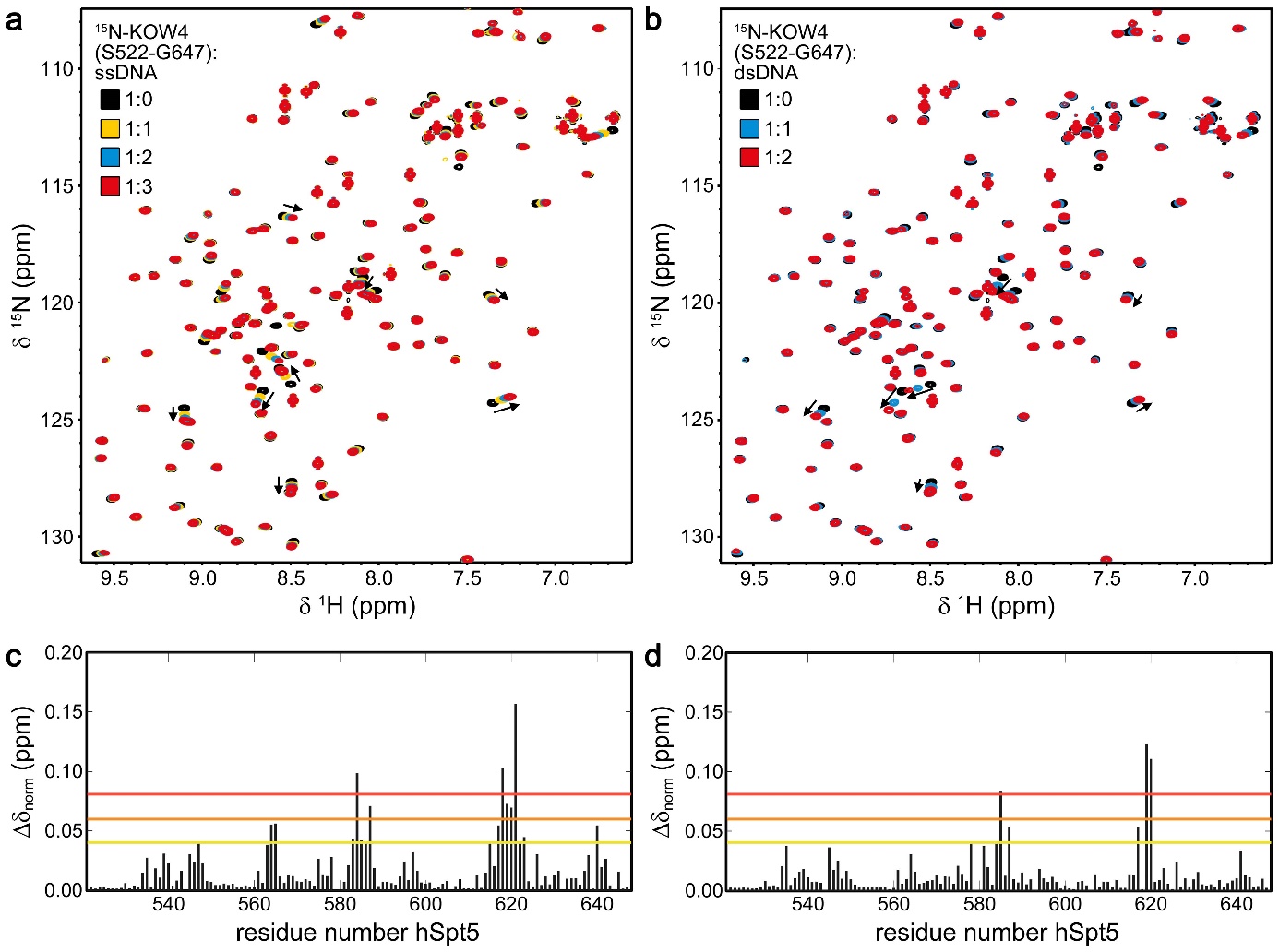
**

**Figure S3: Chemical shift changes of ^15^N labelled KOW4 (S522-G647) upon binding of ssDNA and dsDNA. (a, b)** Overlay of [^1^H, ^15^N] HSQC spectra of 70 µM KOW4 (S522-G647) recorded during titration at different protein: ssDNA ratios: **(a)** 1:0 (black); 1:1 (orange); 1:2 (blue); 1:3 (red) or protein:dsDNA ratios: **(b)** 1:0 (black); 1:1 (blue); 1:2 (red). Relevant chemical shift changes are indicated by arrows. **(c,d)** Normalized chemical shift changes upon ssDNA **(c)** or dsDNA **(d)** binding to KOW4 (S522-G647)**.** Changes larger than 0.04 ppm were considered significant, changes from > 0.04 to 0.06 ppm were assigned as weak (yellow), > 0.06 -0.08 as medium (orange), and > 0.08 as strong (red).

**
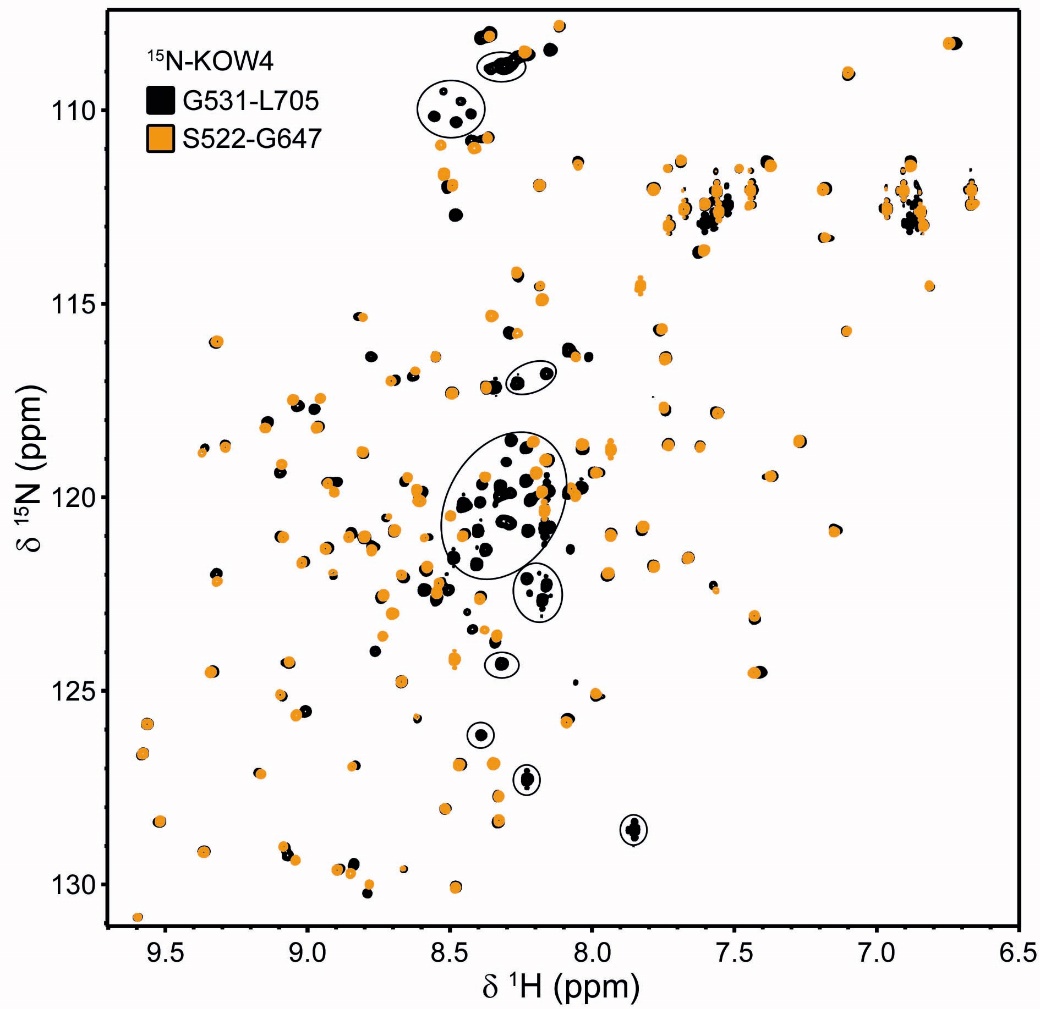
**

**Figure S4: Identification of residues located in the KOW4 – KOW5 linker region**. Overlay of [^1^H, ^15^N] HSQC spectra of 60 µM KOW4 (G531-L705) (black) and 60 µM KOW4 (S522-G647) (orange). Additional signals corresponding to the linker region of KOW4 (G531-L705) are encircled.
